# Supplementary material for: Freely Available, Fully Automated AI-Based Analysis of Primary Tumour and Metastases of Prostate Cancer in Whole-Body [18F]-PSMA-1007 PET-CT
Source: Diagnostics (Basel). 2022 Aug 30;12(9):2101. doi: 10.3390/diagnostics12092101 (PMC9497460; doi:10.3390/diagnostics12092101)
Supplement: Supplementary file 1 [file diagnostics-12-02101-s001.zip › diagnostics-1871168-supplementary.pdf]

Supplementary files

Table S1. Pixelwise encoding for the organ mask. Each pixel encodes 6 different channels (Ch. x) according to the labels given by the automatic segmentation. If the segmentation yields any other label, all channels are encoded as 0.

Organ labels	Ch. 0	Ch. 1	Ch.2	Ch. 3	Ch.4	Ch. 5
Brain	0	0	1	0	0	0
Pancreas, ventricle, liver, spleen	0	0	0	1	0	0
Kidney	0	0	0	0	1	0
Urinary bladder	1	0	0	0	0	1
Bone	1	1	0	0	0	0
Hip Bone	1	1	0	0	0	1
Gastrointestinal tract	1	0	1	0	0	0
Aorta	1	0	0	1	0	0
Lung, Trachea, Bronchi	1	0	0	0	1	0
